# Comparing Platforms for *C. elegans* Mutant Identification Using High-Throughput Whole-Genome Sequencing

**DOI:** 10.1371/journal.pone.0004012

**Published:** 2008-12-24

**Authors:** Yufeng Shen, Sumeet Sarin, Ye Liu, Oliver Hobert, Itsik Pe'er

**Affiliations:** 1 Department of Computer Science, Columbia University, New York, New York, United States of America; 2 Center for Computational Biology and Bioinformatics, Columbia University, New York, New York, United States of America; 3 Department of Biochemistry and Molecular Biophysics, Howard Hughes Medical Institute, Columbia University Medical Center, New York, New York, United States of America; Massachusetts General Hospital/Harvard Medical School, United States of America

## Abstract

**Background:**

Whole-genome sequencing represents a promising approach to pinpoint chemically induced mutations in genetic model organisms, thereby short-cutting time-consuming genetic mapping efforts.

**Principal Findings:**

We compare here the ability of two leading high-throughput platforms for paired-end deep sequencing, SOLiD (ABI) and Genome Analyzer (Illumina; “Solexa”), to achieve the goal of mutant detection. As a test case we used a mutant *C. elegans* strain that harbors a mutation in the *lsy-12* locus which we compare to the reference wild-type genome sequence. We analyzed the accuracy, sensitivity, and depth-coverage characteristics of the two platforms. Both platforms were able to identify the mutation that causes the phenotype of the mutant *C. elegans* strain, *lsy-12*. Based on a 4 MB genomic region in which individual variants were validated by Sanger sequencing, we observe tradeoffs between rates of false positives and false negatives when using both platforms under similar coverage and mapping criteria.

**Significance:**

In conclusion, whole-genome sequencing conducted by either platform is a viable approach for the identification of single-nucleotide variations in the *C. elegans* genome.

## Introduction

Genetically amenable model organisms have been extensively subjected to forward genetic screening approaches in which mutant individuals that are defective in a given biological process are isolated. Mutant isolation has traditionally been followed by time consuming mapping procedures that localize the experimentally induced region to a specific locus. We have previously shown that sequencing with the Genome Analyzer (GA) by Illumina is capable of identifying a molecular lesion in a *C. elegans* strain, *lsy-12(ot177)*, that results in a neuronal cell fate defect, thereby demonstrating the utility of whole-genome sequencing as a quick and cost-effective way to circumvent classic genetic mapping [1]. Disease-causing mutations in a human cancer patient have also recently be reported through whole-genome sequencing [2], illustrating the rising importance of this experimental strategy.

In an effort to better inform the design, implementation and analysis of such genome-wide deep sequencing experiments, we now report sequencing of the same *lsy-12(ot177)* mutant strain, but now using another platform, SOLiD by ABI [3]. We compare these parallel datasets, putting special emphasis on a 4 MB interval around the functional mutation where we have validated the discovered variants using lower throughput Sanger re-sequencing.

## Results

We sequenced genomic DNA samples, isolated from the *C. elegans lsy-12(ot177)* mutant strain [1], [4], with the SOLiD and GA platforms. Both SOLiD and GA runs provided us with similar amounts of raw sequence. In order to separate issues directly related to the sequencing platforms from those pertaining to mapping reads to the genome, we used the same mapping tool, Maq [5] for both platforms, but also used the vendor-provided alignment tool, corona-lite by ABI (http://solidsoftwaretools.com/gf/).

### Mapping of reads

Both SOLiD and GA reads were produced in paired-ends, with SOLiD reads at a size of 25 bp and GA reads at a size of 35 bp. Totally 146 million SOLiD reads were mapped to the wild-type reference genome in total, representing an average depth-coverage of 33× (Table 1). Based on the library preparation protocol as well as the mapping result, we define a pair of reads as good if the two are mapped with correct order and orientation, and the distance between them is less than 5000 bp for SOLiD and 500 bp for GA. Among the mapped SOLiD reads, 82.6 million (57% of mapped) were mapped in good pairs, and 37.3 million reads (26% of mapped) were mapped as single ends, i.e., the other read of the pair was not mapped. 10.9 million pairs (15% of mapped reads) of reads were mapped to different chromosomes. In comparison, 91% of mapped GA reads were in good pairs, 2.6% in single ends, and only 0.4% were mapped to different chromosomes. Nevertheless, the average coverage of good reads (defined as reads that are (a) mapped with no more than 3 mismatches (see Methods), and (b) either in good pairs or single ends) from both platforms are almost identical at about 25×, as summarized in Table 1.

**Table 1:** Sequencing and mapping statistics.

Platform	SOLiD by Maq	GA by Maq	SOLiD by corona-lite
Read size	25 bp	35 bp	25 bp
Total reads (million)	256	125	256
Mapped (million reads/Gb)	146/3.65	84.5/2.96	109/2.73
Good pairs (million)/percentage	41.3/57%	38.6/91%	35.5/65%
Single end mapped (million)/percentage	37.3/26%	2.23/2.6%	35.2/32.6%
Avg. depth-coverage	33×	28×	27×
Avg. depth-coverage from good reads	25×	25×	NA
Mapped to different chromosomes (million)/percentage	21.9/15%	0.3/0.4%	NA

### Depth-coverage

**Figure 1:**
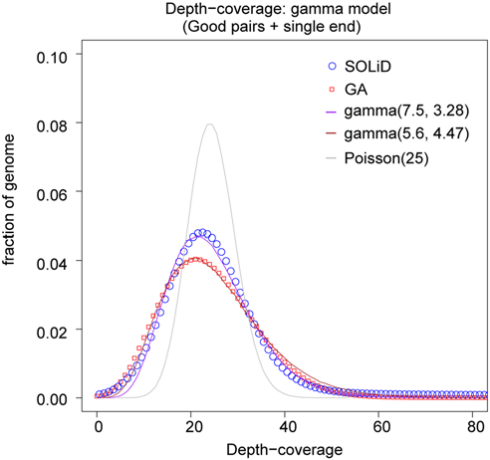
Distribution of depth-coverage. The distribution of depth-coverage of the entire genome is shown for both SOLiD and GA. Poisson and gamma distributions with comparable average mean values are imposed on the observed distribution. Only reads that are mapped with no more three mismatches and without inconsistent mate-pairs are counted in the depth-coverage calculation.

The depth-coverage distributions of the entire genome from both SOLiD and GA sequencing are summarized in Table 2. Both can be modeled as Gamma distributions (Figure 1) [1]. Compared to the Poisson distribution with the same mean value, which was assumed to be the model of depth coverage in some earlier studies [6], these fitted gamma distributions have more weight on both tails.

**Table 2:** Depth-coverage statistics.

Depth-coverage	SOLiD	GA
> = 0	99.98%	99.96%
> = 5	99.65%	99.45%
> = 10	97.71%	95.83%
> = 15	91.64%	86.22%
> = 20	78.58%	70.85%
> = 25	59.19%	52.96%
> = 30	38.79%	36.18%
> = 100	1.56%	0.383%

### Single Nucleotide Variants

7385 variants were called from GA, and 5798 were called from SOLiD. We considered mapping errors and sequencing errors to improve the accuracy of variant calling. Specifically, we only considered variants that meet the following conditions:

1. There are at least two reads from both strands that contain the variant allele. Any lower threshold significantly increases the number of reported variants that are likely false positives, without adding many true positives (see below).
2. The average number of hits per read in the position is less than 1.1. This represents a conservative cutoff to avoid repeats and alleviate the mapping issues with the shorter SOLiD reads.
3. The depth-coverage is less than 60. That is, we filtered out variants with >60× variants, as those are suspected to lie within repeat regions.
4. GA reads only: The number of reads representing the wild-type allele is less than the number of reads representing the variant allele. This condition is based on our previous analysis validation of the GA genome dataset [1].

After such filtering, 901 total genomic variants were left for SOLiD and 1094 variants for GA. 685 of them were shared by both platforms (Table 3). We previously reported that within a 4 Mb region on chromosome V (into which the *lsy-12* mutant was mapped), GA sequencing detected 32 validated variants and 16 false-positive variants [1] (Table 4). With the filtering criteria mentioned above, the 16 false-positive variants were reduced to 4. However, the filtering criteria also eliminated a true, validated variant, leaving 31 variants. SOLiD detected 23 variants, 22 of which were the same as the previously validated ones and one was a new variant (Table 4). This variant was also detected by GA, only with smaller coverage – three reads from one strand and one from the other. We conducted Sanger sequencing on this location and confirmed that this variant (an intergenic variant at position 7953203 on chrV) is genuine. Among the 9 confirmed variants missed by SOLiD, 5 were detected with less than 2 reads on at least one of the strands. This reflects insufficient depth-coverage. The average depth-coverage at the chrV 4 Mb region approximately matches the genome-wide average from both SOLiD and GA sequencing. However, the SOLiD reads are shorter, so SOLiD reads covering true variants are more likely to be rejected by the mapping process because the maximum allowed mismatches are fixed at 3 for both SOLiD and GA (see Methods). The depth-coverage of the variant bases from SOLiD is therefore lower than that from GA. Two other false negatives are due to the fact that the average number of hits of the reads covering the variant base is larger than 3.0, which is a strong indication of a multiple-copy repeat. Another false negative was due to incorrect mapping of SOLiD reads probably because of the way Maq treats SOLiD's two-color encoding. This site appears to be detectable by corona-lite with the same filtering rules. The last false negative was missed due to absence of reads mapping to the site. Trading off specificity for sensitivity, i.e., requiring a single read on one strand and two on the other, detects three additional true variants, but also 11 suspected false positives not reported by GA.

**Table 3:** Variants.

	SOLiD	GA	Common variants (confirmed true/confirmed false/repeats)
Raw	5798	7385	1689 (NA/NA/559)
Filtered	901	1094	685 (NA/NA/0)
ChrV 4 Mb region, raw	180	180	42 (32†/1/9)
ChrV 4 Mb region, filtered	24	35	23 (23†/0/0)

† Variants listed in Table 4.

**Table 4:** Experimentally validated single nucleotide variants.

Position on chromosome V	GA Variants called in Ref. [1]	Found by SOLiD	Why not found by SOLiD?	Type of variant 1
6302463	YES	NO	Repeats	non-exonic
6889636	YES	NO	Low coverage	exonic, silent
6889637	YES	NO	Low coverage	exonic, amino-acid changing
6956711	YES	NO	Low coverage	exonic, amino-acid changing
6956743	YES	NO	Low coverage	exonic, amino-acid changing
6956744	YES	NO	Low coverage	exonic, amino-acid changing
7245105	YES	YES		non-exonic
7377580	YES	YES		non-exonic
7403427	YES	YES		exonic, silent
7430567	YES	YES		exonic, amino-acid changing
7524635	YES	YES		non-exonic
7546600	YES	YES		exonic, amino-acid changing
7860248	YES	NO	Repeats	non-exonic
7953203	NO	YES		non-exonic
8101405	YES	NO	Mapping	non-exonic
8571627	YES	YES		exonic, amino-acid changing
8646873	YES	YES		non-exonic
8657771	YES	YES		non-exonic
8758179	YES	YES		exonic, amino-acid changing
9059200	YES	YES		non-exonic
9217870	YES	YES		non-exonic
9218397	YES	YES		non-exonic
9245971	YES	YES		exonic, amino-acid changing
9376379	YES	YES		exonic, amino-acid changing
9662867	YES	NO	Not covered	non-exonic
9663159	YES	YES		non-exonic
9707449	YES	YES		non-exonic
9846725 *(lsy-12)* 2	YES	YES		exonic, amino-acid changing
9927293	YES	YES		non-exonic
9928614	YES	YES		exonic, silent
9986752	YES	YES		exonic, silent
10234234	YES	YES		non-exonic
10397711	YES	YES		non-exonic

1 The nucleotide change of the variants are shown in [1]. “Non-exonic” is either intergenic or intronic. The one new variant identified by SOLiD is a C to T substitution.

2 This is the variant that is responsible for the mutant phenotype of *lsy-12* animals [1].

The causal mutation in the *lsy-12(ot177)* strain, a G to A nonsense mutation in the predicted gene R07B5.9 [1] was detected by both GA and SOLiD under the filtering rules described above (Table 3).

Without any filtering, both platforms identified about 180 raw variants in the 4 Mb region with 42 shared. Among the shared ones, 32 were confirmed by Sanger sequencing, 9 were left unvalidated, and 1 was confirmed false. 8 of the unvalidated variants and 1 confirmed false were from repeat regions, as indicated by the average number of hits per read (>1.1). The other unvalidated variant might be false positive because it appears to be heterozygous. It is notable that, in total, 16 variants from GA were confirmed to be false in our previous study, yet only one false-positive was shared between datasets [1]. This indicates that most of the raw variants detected by both platforms are genuine except those in repetitive regions.

### Indels

We called small insertions and deletions (indels) using Maq. Similar to variant filtering, we discarded indels in consideration of mapping errors and sequencing errors. We designed two sets of rules:

1. Normal filtering:
  1. coverage <80, and
  2. number of indel reads from each strand >1.
2. Liberal filtering:
  1. coverage <80, and
  2. number of indel reads from each strand > = 1.

The result of indels is summarized in Table 5. 618 indels were shared between normal/GA and liberal/SOLiD. If we assume that the indels shared between two platforms are mostly genuine, the result indicates that indels from the SOLiD sequencing are more likely to be true and require less stringent filtering. This is consistent with the results from our variant analysis.

**Table 5:** Indels.

# of indels	SOLiD	GA	common
normal filtering	420	1280	374
liberal filtering	782	1796	663

From the chrV 4 Mb region, 26 indels were reported in our previous study of the GA sequence run [1]. We get 17 of them from SOLiD with normal filtering. With the liberal filtering rules described above, we get 22 indels, among which 19 were validated by manual resequencing. The remaining three were left unvalidated.

For the GA, with the normal filtering rules described above, we get 29 indels, 25 of which were validated by manual re-sequencing, 4 were left unvalidated. One indel published was missed here due to low coverage from one DNA strand. With liberal filtering, we get all 26 confirmed indels and an additional 14 indels which were left unvalidated.

### Sequencing Errors

The SOLiD technology has built-in error-detection and correction. The corona-lite mapping tool (http://solidsoftwaretools.com/gf/) therefore provides separate statistics regarding observed mismatches between reads and the genome sequence. These include 70 million automatically correctable “single” mismatches, and 2.8×10^6^ “adjacent invalid” mismatches that are detectable, even if not unambiguously correctible errors. Errors that escape these filters make up the actual inaccuracy of the system. These, along with genuine variants, make up the 10^6^ “adjacent valid” mismatches. 93–97% of this group is likely due to errors, based on two consistent estimates: The count of “adjacent invalid” mismatches is expected to be triple the total number of “adjacent valid” mismatches due to errors; also, the estimated 10^3^ genuine variants are expected to incur 30,000–40,000 “adjacent valid” mismatches. These 10^6^ errors among the mapped reads reflect an error rate of 0.036%. Similarly, there are 16×10^6^ mismatches reported from GA reads that were mapped in good pairs. This represents an error rate of 0.6%.

## Discussion

We compared here the performance of detecting mutations from forward genetics by two high-throughput platforms. We were able to find the causal mutation by both SOLiD and Solexa at similar average coverage. The SOLiD reads are relatively shorter. This is likely the main reason for a lower fraction of mapped reads in good pairs, and a reason for a larger fraction of the genome being covered by more than 100× reads; this fraction mostly includes regions with repeating 25-mers.

In the chrV 4 Mb region in which we had mapped *lsy-12*, we detected one new variant by SOLiD sequencing, which we validated by Sanger sequencing. It was also present in raw Solexa reads but was discarded due to low coverage. However, SOLiD also missed a substantial number of validated variants that GA correctly called. In the entire genome the number of non-repeat raw variants detected by both SOLiD and GA is 1130 (Table 3), which is close to the number of variants detected by either platform after filtering. This suggests that the majority of the non-repeat variants detected by both platforms are genuine, and the systematic sequencing errors from these two platforms are caused by different sources.

**Figure 2:**
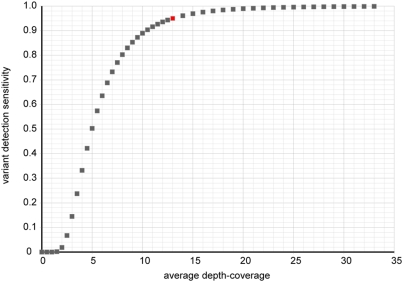
The relationship between average depth-coverage and variant detection sensitivity. The x-axis is the average depth-coverage. The y-axis is the theoretical fraction of genome where potential variants can be detected under the assumptions described above. The red dot marks 95% sensitivity at 13× coverage.

Practically, it is important to further reduce the cost of sequencing while keep reasonable sensitivity and accuracy of mutation detection. Given the instrument and protocol, one way to do this is to reduce the overall depth-coverage to a minimum level. Based on our result, having at least two reads from both strands is a good basic measurement of variants. If we assume that the depth-coverage follows gamma distribution, Gamma(6, *C*/6), where *C* is the average coverage, and the sampling of two strands follow simple Binomial distribution B(n, 0.5), then it is possible to calculate the theoretical relationship between *C* and the proportion of genomic region where at least two reads from each strand cover, and assuming the sequencing is error-free and the genome does not contain repeats that could hinder mapping. The result is summarized in Figure 2. In order to achieve 95% sensitivity, 13× is required based on this calculation. Under current protocol, roughly half a run produces 13× mappable GA or SOLiD reads. This is different from the discussion in our previous paper [1] that took a more liberal filtering approach, that would require more re-sequencing for validation. 13× coverage aims at sampling of two strands and the requirement of having at least two variant-containing reads from both strands, with very few false positives.

In conclusion, we found that SOLiD calls less false-positives variants compared to the GA and GA calls less false-negatives variants compared to the SOLiD. The tradeoff between tolerating false negatives, and being able to follow up false candidates is therefore an important determinant for platform choice (summarized in Table 6). In our specific example of mutant identification in the *C. elegans* genome, the experimental system allows narrowing down the region of interest to a fraction of the genome by traditional mapping and the system allows the following-up of dozens of variants by various experimental strategies (including sequencing multiple alleles, rescue analysis, RNAi analysis etc.; [1]). A false negative, i.e. the missing of the one phenotype-causing mutation, is not tolerable; therefore, the GA platform appears the preferable choice for our system. Another important consideration in choosing between the GA or SOLiD approach is the effort required for preparing the DNA library to be sequenced. It has been previously noted that the emulsion PCR step required for the SOLiD platform is cumbersome and technically challenging [3], which contrasts the apparently straight-forward library preparation step for the GA. Whatever platform one uses, it is clear that whole-genome sequencing may revolutionize forward genetic analysis in model organisms such as *C. elegans*.

**Table 6:** Comparing platforms.

Feature	Preferred Platform
Reducing false positives	SOLiD
Reducing false negatives	GA
Raw accuracy	SOLiD
Mapping	GA
Ease of library preparation	GA

We emphasize that this work represents only a snapshot-comparison undertaken during a technological tornado. Multiple vendors, including those discussed here, but others as well [3], continue to push the envelope in terms of sequence accuracy, read-length (and therefore mapping) as well as affordable throughput. When planning future sequencing work one therefore needs to take evidence for deficiencies in past performance with a grain of salt. Still, as the challenges for sequencing continue to grow hand in hand with the boundaries of feasibility, we believe that the principles and considerations described in this manuscript will be of use even when our exact numbers will have become obsolete.

## Materials and Methods

### DNA sample preparation

Genomic DNA preparation: Genomic *C. elegans* DNA was prepared using a modified protocol obtained from the Comprehensive Protocol Collection at Dartmouth University (http://www.dartmouth.edu/~ambros/protocols/MGH_protocols/Worm_genomic_DNA.html). Forty 5-cm plates of *lsy-12* mutants worms were used. DNA concentrations were estimated using agarose gel electrophoresis. 5 µg were provided to Illumina's sequencing service, as previously reported [1] and 100 µg were provided to Agencourt, who performed the ABISolid runs.

### DNA Sequencing

Sequencing runs were performed by Agencourt Bioscience Corporation (a Beckman Coulter Company) for the ABI Solid runs and by Illumina's in-house sequencing services for the GA sequence run, as described in [1].

### Bioinformatic analysis

All reads were mapped using Maq. The maximum allowed number of mismatches per read was 3 for both platforms. This cutoff was selected to accommodate both mismatches due to true variants, as well as ones due to errors that at are rare per bp, but much more frequent per-read. The maximum outer distance for a correct read pair was set to 5000 for SOLiD and 250 for GA. Other parameters were default. The SOLiD reads were treated slightly different than GA reads in Maq: the -p in pileup function is supposed to output only the read pairs that are regarded as good, but none of the SOLiD pairs are regarded good because SOLiD reads are always FF or RR oriented, whereas only FR reads are regarded as good reads – based on the man page on mapview function. So we took all good paired SOLiD reads as well as single-end mapped reads and re-mapped them using Maq. As for GA reads, we output pileup using -p option. Thus the only difference is the single-ended reads are discarded in GA pileup, which would not have much impact on the variant and coverage analysis because they only contributed 2.6% to all mapped GA reads.
